# Mesenchymal stem cell-derived extracellular vesicles as probable triggers of radiation-induced heart disease

**DOI:** 10.1186/s13287-021-02504-5

**Published:** 2021-07-22

**Authors:** Lan Luo, Chen Yan, Naoki Fuchi, Yukinobu Kodama, Xu Zhang, Goto Shinji, Kiyonori Miura, Hitoshi Sasaki, Tao-Sheng Li

**Affiliations:** 1 Department of Stem Cell Biology, Nagasaki University Graduate School of Biomedical Sciences, 1-12-4 Sakamoto, Nagasaki, 852-8523 Japan; 2 Medical Technology School of Xuzhou Medical University, Xuzhou Key Laboratory of Laboratory Diagnostics, Tongshan Road 209, Xuzhou, 221004 China; 3 Department of Obstetrics and Gynecology, Nagasaki University Hospital, Nagasaki, 852-8523 Japan; 4 Department of Pharmacy, Nagasaki University Hospital, Nagasaki, 852-8523 Japan

**Keywords:** Mesenchymal stem cells, Extracellular vesicles, Ionizing radiation, Heart disease

## Abstract

**Background:**

Radiation-induced heart disease has been reported, but the underlying mechanisms remain unclear. Mesenchymal stem cells (MSCs), also residing in the heart, are highly susceptible to radiation. We examined the hypothesis that the altered secretion of extracellular vesicles (EVs) from MSCs is the trigger of radiation-induced heart disease.

**Methods:**

By exposing human placental tissue-derived MSCs to 5 Gy γ-rays, we then isolated EVs from the culture medium 48 h later and evaluated the changes in quantity and quality of EVs from MSCs after radiation exposure. The biological effects of EVs from irradiated MSCs on HUVECs and H9c2 cells were also examined.

**Results:**

Although the amount and size distribution of EVs did not differ between the nonirradiated and irradiated MSCs, miRNA sequences indicated many upregulated or downregulated miRNAs in irradiated MSCs EVs. In vitro experiments using HUVEC and H9c2 cells showed that irradiated MSC-EVs decreased cell proliferation (*P* < 0.01), but increased cell apoptosis and DNA damage. Moreover, irradiated MSC-EVs impaired the HUVEC tube formation and induced calcium overload in H9c2 cells.

**Conclusions:**

EVs released from irradiated MSCs show altered miRNA profiles and harmful effects on heart cells, which provides new insight into the mechanism of radiation-related heart disease risks.

## Background

In the 1960s, clinicians first recognized that cardiovascular complications occurred in patients who underwent radiotherapy (RT) for chest tumors [1]. Subsequent studies involving patients who received relatively high thoracic RT doses demonstrated an excess risk of radiation-induced heart disease (RIHD) [2–4]. The incidence of RIHD declined considerably with decreased cardiac radiation exposures using modern RT techniques (intensity-modulated RT, image-guided RT, etc.) [5]. Indeed, Darby and van den Bogaard et al. identified that the mean heart doses linearly correlated with the risk of RIHD [5, 6]. However, the minimum threshold dose remained unclear. Epidemiological data revealed increased heart disease risks in atomic bomb survivors with individually estimated doses over 0.5 Gy [7]. In addition, individuals with other traditional risk factors (e.g., hypertension, hyperlipidemia, diabetes mellitus, smoking), or at a young age, seemed to be more vulnerable to developing RIHD [8]. The rising radiation exposure potentiality of medical imaging, galactic cosmic, or terrestrial ionizing resources makes it reasonable to worry about RIHD.

The clinical presentations of RIHD are currently well documented, including coronary artery atherosclerosis, pericarditis, cardiomyopathy, and conduction defects [9]. RIHD usually takes years or decades to manifest, making it challenging to interpret the underlying cellular and molecular mechanisms. Currently, ionizing radiation causes endothelial dysfunction and inflammatory responses, preceding the development of atherosclerosis, cardiac fibrosis, and tissue remodeling [9]. Sustained DNA lesions, oxidative stress, mitochondrial dysfunction, epigenetic regulation, and telomere erosion are also related to the development of RIHD [10–12]. Notably, these molecular changes interact with each other and act diversely in different types of heart cells. Therefore, the pathophysiological mechanisms of RIHD are unclear.

Classical radiobiology identifies that cells with high proliferative rates and immature features are more susceptible to ionizing radiation. Cardiomyocytes, the primary cell type in the heart, are postmitotic and incapable of proliferating. Hence, the dogma heart, as a radioresistant organ, has lasted for a long time. However, apart from cardiomyocytes, other cells such as microvascular endothelial cells, fibroblasts, and recently identified mesenchymal stem cells (MSCs) [13] also reside in the heart. Although there is no consensus on the number of MSCs in various tissues/organs, rare resident MSCs are known to play a critical role in maintaining tissue homeostasis. Following myocardial infarction, cardiac CD45^-^CD44^+^DDR2^+^ MSCs proliferated and exhibited typical characteristics with multipotent differentiation capacity and clonogenic expansion [14]. Previous studies identified that injuries to hematopoietic stem cells (0.01 to 0.05% in the bone marrow) after radiation exposure are acknowledged to contribute to a future increased leukemia risk [15]. We have recently demonstrated that cardiac MSCs are highly radiosensitive [16, 17], and whole-body irradiation with 3 Gy γ-rays impairs the endogenous regeneration of infarcted mouse hearts [18]. Thus, MSCs theoretically reveal higher radiosensitivity than other mature heart tissue cells such as endothelial cells and cardiomyocytes. In response to different stimuli, MSCs release abundant extracellular vesicles (EVs), which are essential mediators of intercellular communication [19]. Hypoxia-primed bone marrow MSCs promoted cardiac function in a mouse model of myocardial infarction via upregulated EV miR-125b-mediated cell protection [20]. Thus, we hypothesize that radiation exposure alters the secretion of EVs from MSCs, which subsequently initiates/triggers the damage of other heart tissue cells with less radiosensitivity.

By exposing human placenta-derived mesenchymal stem cells (hp-MSCs) to 5 Gy γ-rays, we investigated the radiation-induced change in the secretion of EVs from hp-MSCs in this study. We further evaluated the potential role of EVs from irradiated hp-MSCs in regulating the survival and function of heart tissue cells in vitro.

## Methods

### Culture of hp-MSCs, HUVEC, and H9c2 cells

hp-MSCs derived from three donors were obtained as a gift [21]. hp-MSCs were maintained in Dulbecco’s modified Eagle’s medium (DMEM) (Wako, Osaka, Japan) supplemented with 10% fetal bovine serum (FBS, HyClone Laboratories, Logan, UT, USA), 10 ng/ml human recombinant basic fibroblast growth factor (Wako), and 1% penicillin (100 U/ml)/streptomycin (100 U/ml) solution (Life Technologies). The HUVEC cell line was purchased from PromoCell GmbH (Germany) and grown in endothelial cell growth medium 2 (PromoCell) supplemented with 10% fetal bovine serum and 1% penicillin/streptomycin. The H9c2 cell line was purchased from ATCC (CRL-1446) and grown in DMEM supplemented with 10% fetal bovine serum and 1% penicillin/streptomycin. All cells were cultured in a 5% CO_2_ incubator at 37 °C.

### Flow cytometry analysis

When grown to 80% confluence, the twice passaged hp-MSCs (n=3) were harvested with 0.25% trypsin. Then, the cells were washed with phosphate-buffered saline (PBS) (Wako, Osaka, Japan) and centrifuged at 300 g for 3 min. The cell pellets were resuspended in 800 μl of 1% BSA and aliquoted into 8 EP tubes. Cells were then stained with CD44-phycoerythrin (PE) (IM7), CD105-PE (SN6), CD90-FITC (eBio5E10), CD73-FITC (AD2), CD45-PE (HI30), and CD44-phycoerythrin (PE) (IM7). Unstained cells were included as blank controls. Cells stained with respective isotopes were included as a negative control. Flow cytometry analysis was performed using a FACSCalibur (Becton Dickinson, Franklin Lakes, Nj, USA). The acquired data were analyzed using Cell Quest software (Becton Dickinson).

### Radiation exposure and EV isolation

hp-MSCs (passaged 2–5, 3×10^5^ cells) were plated on a 10-cm dish. The next day, the culture medium was aspirated, and the cells were washed with PBS to remove the residual FBS. Fresh culture medium supplemented with 10 ml 10% exosome-depleted FBS (System Biosciences) was added. Then, the hp-MSCs were exposed to 0 or 5 Gy γ-rays at a dose rate of 1 Gy/min using a PS-3100SB γ-ray irradiation system with a Cs source (Pony Industry Co., Ltd. Osaka, Japan) [16].

After 48 h of incubation, culture medium (10 ml) from hp-MSCs that irradiated or not was collected for EV isolation as previously described with minor modifications [22]. Briefly, the culture medium was centrifuged at 300 g for 3 min, at 4 °C and 2000 g for 30 min to remove cell debris and apoptotic bodies. The supernatant was ultracentrifuged at 4 °C and 100,000 g for 120 min to collect EVs. Then, the pellet was washed with PBS and underwent another step of ultracentrifugation at 4 °C and 100,000 g for 120 min to concentrate and purify EVs. Finally, the pellet was resuspended in PBS and passed through a 0.22-μm filter (Millex) for further experiments or stored at −80°C. EVs isolated from a 30-ml culture medium were pooled together as one sample for further experiments.

### EVs imaging and size distribution

The image of EVs (*n*=1) was taken by a transmission electron microscope. Briefly, 5 μl of EVs was dropped on a copper net and incubated at room temperature (RT) for 5 min. Then, excess liquids were removed by filter paper. Five microliters of 1% phosphotungstic acid was added to the copper mesh and incubated for 1 min at RT. Excess liquids were also removed by filter paper. Deionized water was added to the copper mesh to remove excess dye solution. EVs were observed under an electron microscope after drying at RT.

The size distribution of EVs (*n*=1) was estimated by nanoparticle tracking analysis using a Particle Metrix Zeta view.

### EV protein preparation and Western blotting

The protein concentration of EVs (*n*=4) was tested by Micro BCA protein analysis as described in the instructions (Thermo Scientific Pierce 23235). Expression of the EVs markers CD63 and TSG101 (System Biosciences) was verified by Western blot analysis (*n*=1).

### EV miRNA sequences

EVs from three donors of hp-MSCs were pooled for miRNA sequences. EV miRNAs were analyzed using gene chip miRNA 4.0 by Filgen Company. Briefly, EV miRNAs were extracted using the miRNeasy® Serum/Plasma Kit. The extracted miRNAs (150 ng) were further concentrated by Micro Vac^TM^, and their volume was adjusted to 8 μl using nuclease-free water. Then, the hybridization solution was prepared by mixing the hybridization master mix with a biotin-labeled sample according to the manual (FlashTag™ Biotin HSR RNA Labeling Kit for GeneChip™ miRNA Arrays). The array that added hybridization solution was incubated in the GeneChip™ Hybridization Oven 645 for 18 h (48°C, 60 rpm). Later, the array was washed using GeneChip™ Fluidics Station 450. Finally, the array was screened by the GeneChip™ Scanner 3000 7G and analyzed according to the GeneChip™ Command Console (AGCC) 4.0 User Manual.

### Uptake of EVs

EVs (*n*=1) derived from non-irradiated (non-irradiated-EVs) and irradiated hp-MSCs (irradiated-EVs) were labeled with the PKH26 Red Fluorescent Cell Linker Kit (Sigma-Aldrich) according to the manufacturer’s protocol with minor modifications. Non-irradiated or irradiated-EVs diluted in PBS were added to 1 ml diluent C (Sigma-Aldrich). In parallel, 4 μl PKH26 dye was added to 1 ml diluent C and incubated with the EV solution (10 μg/ml) for 4 min. To bind excess dye, 2 ml 0.5% BSA/PBS was added. The labeled EVs were washed at 100,000 g for 1 h, and the EV pellet was diluted in 100 μl PBS and used for uptake experiments. PKH26 labeled non-irradiated or irradiated-EV were cultured with the HUVEC and H9c2 cell lines, respectively. Images of EV uptake were taken after coculture at 3 and 24 h using confocal microscopy.

### Evaluation of cell proliferation and DNA damage

To evaluate the effects of EVs on cell proliferation and DNA damage, HUVEC (4×10^4^) and H9c2 cells (4×10^4^) were seeded on 4-well chamber culture slides. After 72 h of culture with 10 μg/ml non-irradiated-EVs or irradiated-EVs (*n*=3), the cells were washed with PBS and fixed in 4% paraformaldehyde for 10 min. After the incubation of protein block serum-free (DAKO) with 0.01% Triton X-100, the cells were incubated with Ki67 monoclonal antibody (SolA15, Invitrogen), anti-53BP1 antibody (ab36823, Abcam), or anti-gamma H2A.X (ab2893, Abcam), followed by associated Alexa flour 488-conjugated second antibody. Nuclei were stained with DAPI, the positively stained cells were counted under fluorescence microscopy at 200-fold magnification, and 20 fields per section were randomly selected for quantitative counting. The percentage of positive cells in each field was calculated as (positively stained cells/all cells in the field)×100%.

### Annexin-V flow cytometry

To evaluate the effects of EVs on HUVEC and H9c2 cell apoptosis, HUVEC (3×10^5^) and H9c2 (3×10^5^) cells were seeded on 10-cm culture dishes. After 48 h of culture with 10 μg/ml non-irradiated-EVs or irradiated-EVs (*n*=3), the cells were collected and washed with cold D-PBS by centrifugation for 5 min at 500×*g* at 4°C. Cells treated with 3% formaldehyde in a buffer for 30 min were included as a positive control. The cell pellets were suspended with 100 μl cold D-PBS and then added 5 μl of Annexin V-FTIC solution, and 2.5 μl dissolved PI were added as described in the manual (Beckman Coulter). The samples were kept on ice and incubated for 10 min in the dark. Finally, 400 μl ice-cold 1× binding buffer was added to the samples for further experiments. Flow cytometry analysis was performed using a FACSCalibur (Becton Dickinson, Franklin Lakes, NJ, USA). The acquired data were analyzed using Cell Quest software (Becton Dickinson).

### Tube formation

Corning® Matrigel® Matrix (356230) was thawed overnight on the ice at 4°C according to the guidelines in the manual. All pipets and procedures were previously kept on ice. Then, 289 μl chilled Corning Matrigel® matrix into 24-well culture plates to avoid air bubbles. Plates were incubated at 37°C for 30–60 min. The medium remaining was removed carefully without disturbing the matrix layer, and the plates were ready to use. HUVEC cells were previously cocultured with 10 μg/ml non-irradiated-EVs or irradiated-EVs for 48 h (*n*=3). Then, 300-μl cell suspensions were collected and added to each well and incubated at 37°C, in a 5% CO_2_ atmosphere. Tube formation was observed 3 h later under microscopy at 100-fold magnification and 10 fields per section were randomly selected for quantitative counting. The photo was further analyzed by ImageJ.

### Intracellular calcium detection

Intracellular calcium was examined by loading H9c2 cells with Fluo 3 (Dojindo Molecular Technologies, Inc.) according to the instructions. Briefly, 3×10^5^ H9c2 cells were previously seeded on 10-cm culture dishes and cocultured with 10 μg/ml non-irradiated-EVs or irradiated-EVs for 48 h (*n*=3). Cells were harvested and then plated on 96-well plates. The culture medium was carefully removed without injuring the cells. Cells were washed gently with PBS and then incubated with loading buffer at 37°C for 1 h. Loading buffer was removed carefully, and a warm recording medium was added. Finally, the fluorescence was examined by a multifunctional microplate detector.

### Statistical analysis

All the results are presented as the mean ± SD. The statistical significance was determined by one-way ANOVA followed by Turkey’s multiple comparisons test (GraphPad Prism). Differences were considered significant when *P* < 0.05.

## Results

### Characterization of hp-MSCs and hp-MSC EVs

**Fig. 1:**
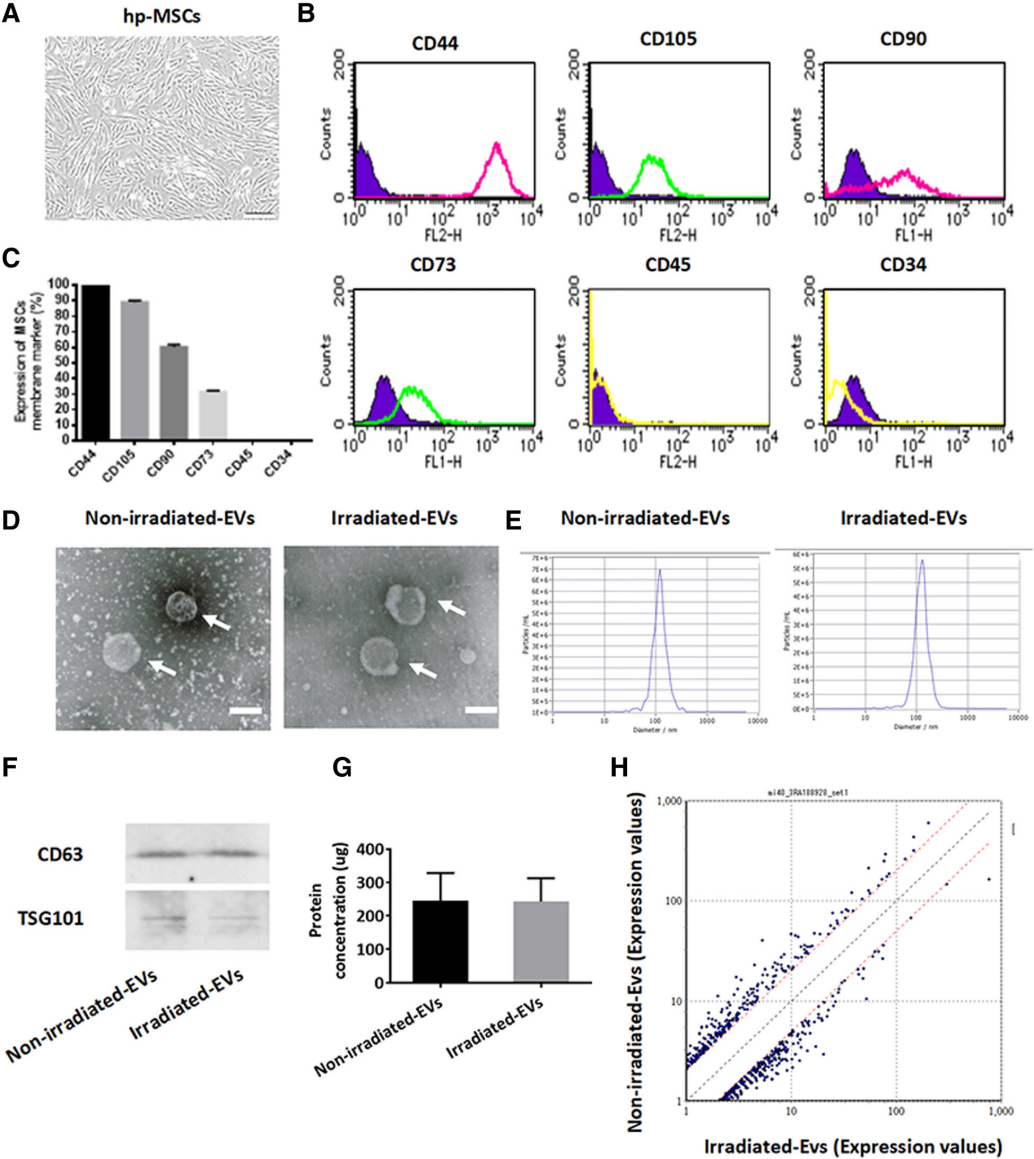
Characterization of hp-MSCs and hp-MSC EVs. **A** Human placental tissue-derived mesenchymal stem cells (hp-MSCs) displayed identical fibroblast morphology. Representative images are shown. Scale bar: 200 μm. Representative histograms (**B**) and quantitative data (**C**) of flow cytometry analysis of the expressions of CD44, CD105, CD90, and CD73, but not CD45 and CD34 in hp-MSCs from two passages. **D** Representative images from electron microscopy show EVs (white arrow) from non-irradiated and irradiated hp-MSCs (*n*=1). Scale bar: 100 nm. **E** Nanoparticle track analysis of the size-distribution of EVs from non-irradiated and irradiated hp-MSCs (*n*=1). **F** Western blotting analysis of CD63 and TSG101 expression in EVs from non-irradiated and irradiated hp-MSCs (*n*=1). **G** Protein concentration of EVs from non-irradiated and irradiated hp-MSCs determined by Micro BCA protein assay. **H** EVs from three donors of hp-MSCs were pooled for miRNA sequences. Scatter plot image indicated miRNAs that were upregulated or downregulated more than two-fold in EVs from non-irradiated and irradiated hp-MSCs. The axis values indicate the expression values after normalization of miRNAs. Values are the mean ± SD. Non-irradiated-EVs: EVs isolated from conditioned medium of non-irradiated hp-MSCs, Irradiated-EVs: EVs isolated from conditioned medium of irradiated hp-MSCs

Primarily expanded hp-MSCs exhibited a fibroblast-like morphology (Fig. 1A) and were identified as the biological properties of MSCs according to their expression pattern on the cell surface markers CD44, CD105, CD90, CD73, CD45, and CD34 (Fig. 1 B, C). To investigate the impact of IR on the EVs secretion, hp-MSCs (passaged 2–5) were exposed to 5 Gy γ-rays and the medium was collected 48 h later for EVs isolation by ultracentrifugation. The successful isolation of EVs was confirmed by electron microscopy (Fig. 1 D), nanoparticle track analysis (Fig. 1E), and Western blot analysis of the expression of membrane markers of CD63 and TSG101 (Fig. 1F). The size distribution (Fig. 1E) and protein concentration (Fig. 1G) were not obviously different between the EVs from the non-irradiated and irradiated MSCs. These data indicated very limited changes in the amount and size distribution of EVs from hp-MSCs within 48 h after exposure to 5 Gy γ-rays.

### EVs from non-irradiated and irradiated hp-MSCs exhibited different miRNA expression profiles

We further measured the expression of miRNAs in EVs by gene chip miRNA 4.0. In contrast to the small changes in the secretion amount and size distribution, the analysis of miRNA sequences indicated that many miRNAs in irradiated MSC-EVs were upregulated or downregulated more than two-fold relative to the levels of the miRNAs of non-irradiated hp-MSCs (Fig. 1H). The top 20 miRNAs of upregulation (Table 1) or downregulation (Table 2) in EVs from irradiated hp-MSCs were listed in the table. Only miR-4655-5p was upregulated over three-fold, but miR-4443, miR-7110-5p, miR-520g-3p, miR-382-5p, miR-424-3p, miR-3197, and miR-6824-5p were downregulated over three-fold in irradiated hp-MSC EVs.

**Table 1:** The top 20 miRNAs that were upregulated in EVs from irradiated hp-MSCs (versus non-irradiated hp-MSCs)

miRNA	Ratio: irradiated-EVs to non-irradiated-EVs	Irradiated-EVs (expression values)	Non-irradiated-EVs (expression values)
hsa-miR-4655-5p	3.20	15.08	4.71
hsa-miR-6506-5p	2.92	9.08	3.11
hsa-miR-4635	2.89	13.53	4.68
hsa-miR-129-5p	2.81	7.13	2.54
hsa-miR-6772-3p	2.81	6.10	2.17
hsa-mir-3689b	2.81	10.45	3.72
hsa-miR-212-5p	2.71	8.65	3.20
hsa-miR-3157-3p	2.60	8.48	3.26
hsa-miR-3120-5p	2.35	6.55	2.78
hsa-miR-16-1-3p	2.35	7.59	3.24
hsa-miR-4638-5p	2.32	7.65	3.29
hsa-miR-2392	2.32	6.18	2.67
hsa-miR-4330	2.31	4.98	2.16
hsa-miR-3652	2.26	7.15	3.17
hsa-miR-4686	2.22	6.34	2.86
hsa-miR-7850-5p	2.22	5.42	2.44
hsa-miR-324-3p	2.15	20.76	9.65
hsa-miR-4535	2.15	12.89	6.01
hsa-miR-4538	2.13	13.57	6.36
hsa-miR-5087	2.12	9.64	4.55

*Non-irradiated-EVs* EVs isolated from conditioned medium of non-irradiated hp-MSCs *Irradiated-EVs* EVs isolated from conditioned medium of irradiated hp-MSCs

**Table 2:** The top 20 miRNAs that were downregulated in EVs from irradiated hp-MSCs (versus non-irradiated hp-MSCs)

miRNA	Ratio: irradiated-EVs to non-irradiated-EVs	Irradiated-EVs (expression values)	Non-irradiated-EVs (expression values)
hsa-miR-4443	0.25	10.98	44.66
hsa-miR-7110-5p	0.26	55.08	212.58
hsa-let-7a-5p	0.28	2.15	7.66
hsa-miR-520g-3p	0.29	6.96	23.87
hsa-miR-382-5p	0.30	5.37	18.20
hsa-miR-424-3p	0.30	8.59	28.78
hsa-miR-3197	0.31	6.71	21.56
hsa-miR-6824-5p	0.33	4.81	14.62
hsa-miR-3178	0.34	204.21	600.47
hsa-miR-32-3p	0.35	3.96	11.39
hsa-miR-1273g-3p	0.35	352.65	1008.29
hsa-miR-23a-5p	0.35	4.19	11.93
hsa-miR-29a-3p	0.36	8.09	22.58
hsa-miR-3663-5p	0.36	7.25	20.14
hsa-miR-146a-5p	0.36	1.65	4.56
hsa-miR-517a-3p	0.38	3.51	9.29
hsa-miR-517b-3p	0.38	3.51	9.29
hsa-miR-3190-5p	0.38	3.25	8.50
hsa-miR-516b-5p	0.38	6.76	17.66
hsa-miR-4692	0.38	2.08	5.42

*Non-irradiated-EVs* EVs isolated from conditioned medium of non-irradiated hp-MSCs *Irradiated-EVs* EVs isolated from conditioned medium of irradiated hp-MSCs

### Uptake of EVs by HUVEC and H9c2 cells

**Fig. 2:**
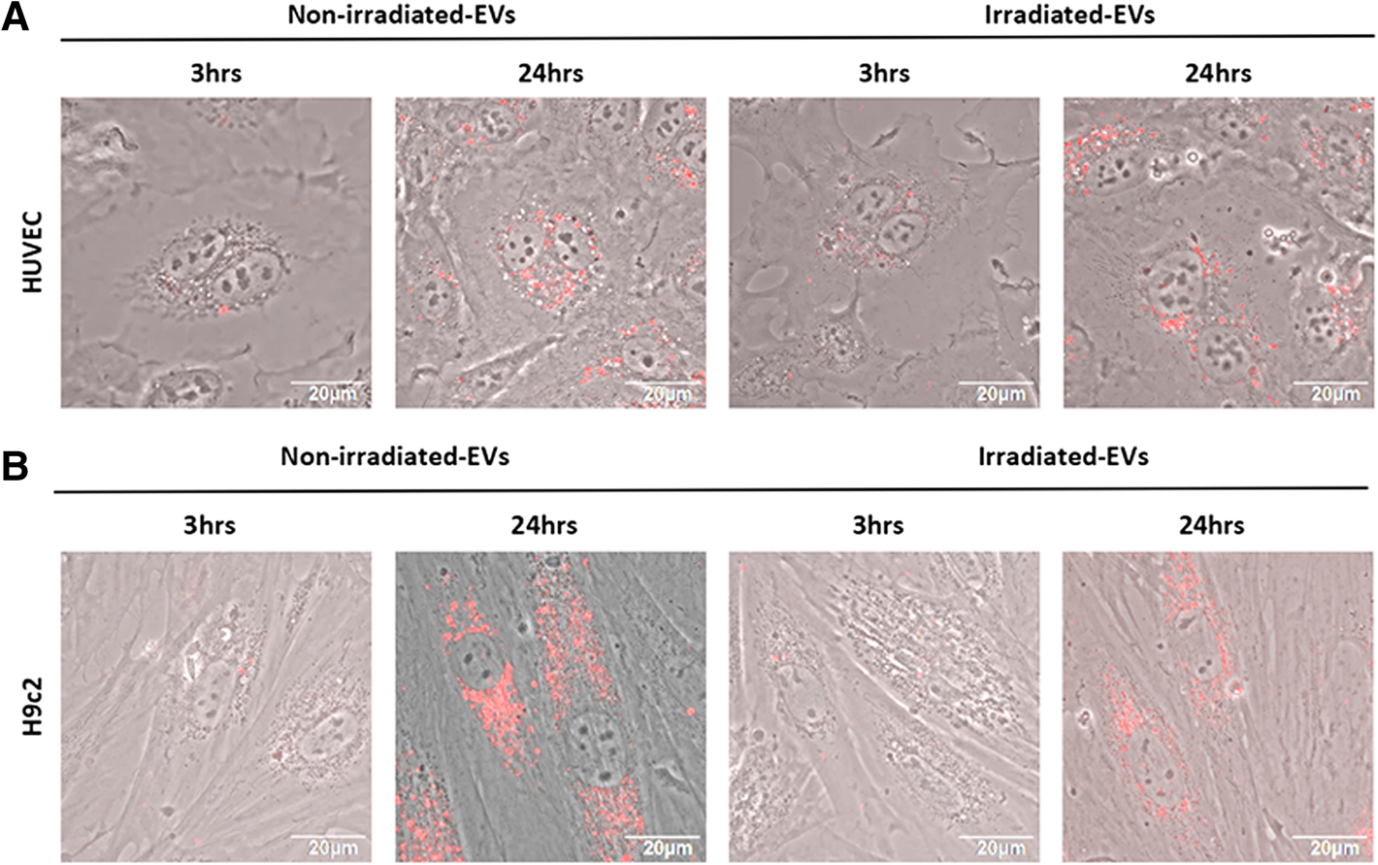
The uptake of hp-MSC-derived EVs by HUVEC and H9c2 cells. Confocal images of HUVEC (**A**) and H9c2 cells (**B**) with internalized PKH26 labeled EVs from non-irradiated/irradiated hp-MSCs after 3 or 24 h of coculture (*n*=1). Scale bar: 20 μm

Then, we evaluated the biological effects of EVs from the non-irradiated and irradiated hp-MSCs on endothelial cells and cardiomyocytes. By culturing HUVEC and H9c2 cells with the supplement of PKH26-labeled EVs (10 μg/ml) in the medium, the uptake of EVs by cells was observed using a confocal microscope. Red fluorescence was clearly detectable in the cytoplasm at 3 h and further enhanced after 24 h (Fig. 2 A, B). However, the uptake of EVs from either non-irradiated or irradiated hp-MSCs was quite similar by HUVEC and H9c2 cells, demonstrating that EVs from non-irradiated or irradiated hp-MSCs could be internalized by the HUVEC and H9c2 cells.

### EVs from irradiated hp-MSCs significantly impaired the survival of HUVEC and H9c2 cells

**Fig. 3:**
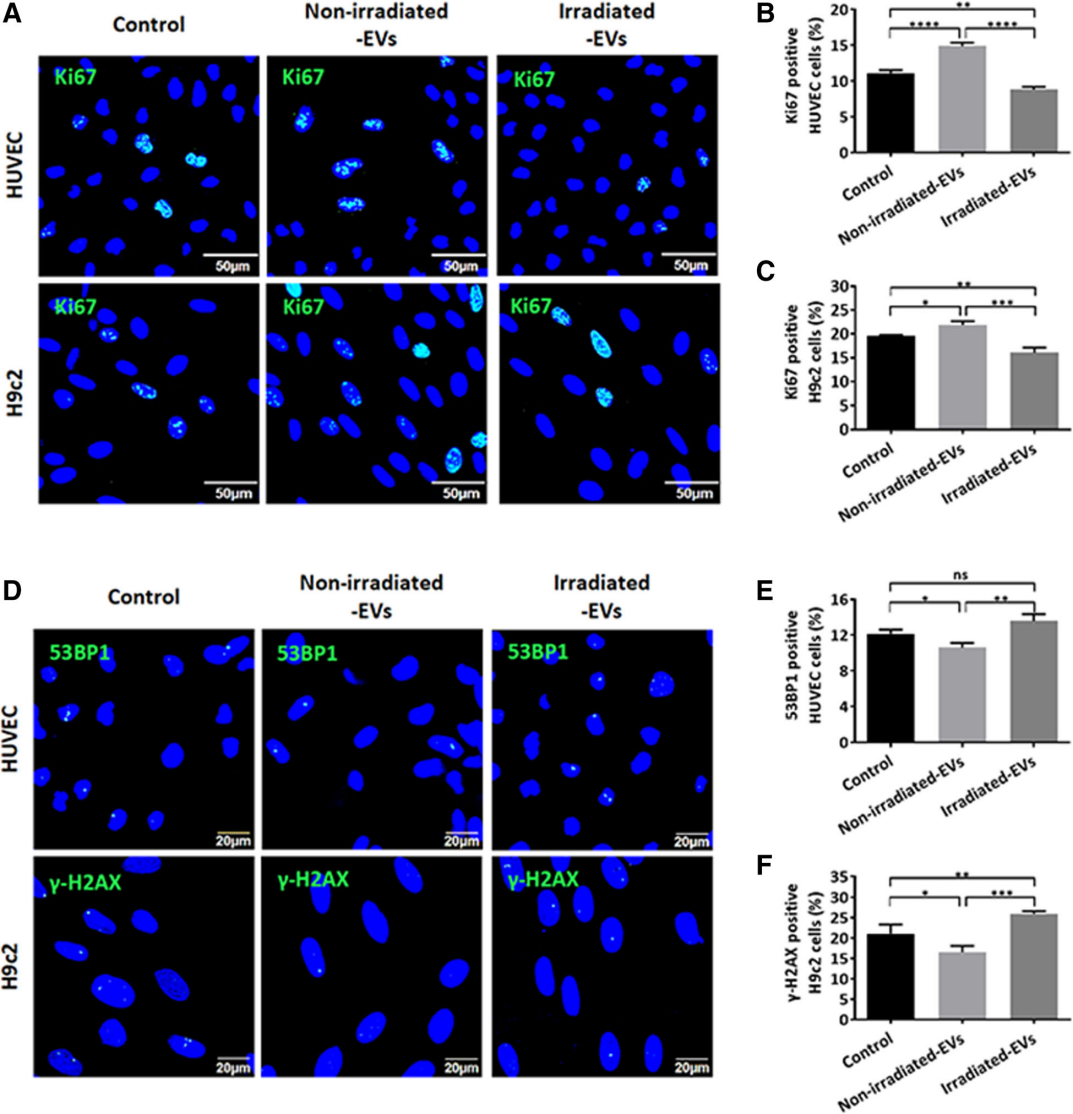
Effects of hp-MSC-derived EVs on the proliferation and DNA damage of HUVEC and H9c2 cells after 72 h of culture. **A** Representative images of immunofluorescence analysis show Ki67-positive HUVEC or H9c2 cells. Scale bar: 50 μm. Quantitative data (*n*=3) indicate the percentage of Ki67-positive HUVEC cells (**B**) or H9c2 cells (**C**). **D** Representative images of immunofluorescence analysis show the DNA damage in HUVEC (53BP1 foci) or H9c2 (γ-H2AX foci) cells. Scale bar: 20 μm. Quantitative data (*n*=3) on the percentage of 53BP1-positive HUVEC cells (**E**) and γ-H2AX-positive H9c2 cells (**F**). Non-irradiated-EVs: EVs isolated from conditioned medium of non-irradiated hp-MSCs, Irradiated-EVs: EVs isolated from conditioned medium of irradiated hp-MSCs. Values are the mean ± SD. ns *P* > 0.5, *P < 0.5, **P < 0.1, ***P < 0.01, ****P < 0.001

The impact of non-irradiated and irradiated hp-MSC EVs on cell proliferation and DNA damage was observed after 72 h of coculture using an immunofluorescence assay (Fig. 3A, D). Ki67 expression in HUVEC and H9c2 cells was increased by non-irradiated hp-MSC EVs but decreased by irradiated hp-MSC EVs (Fig. 3B, C). Due to the different specificities of antibodies between HUVEC and H9c2 cells, DNA damage in cells was detected by the formation of 53BP1 or γ-H2AX foci in nuclei. EVs from non-irradiated hp-MSCs significantly decreased the percentages of cells with 53BP1 or γ-H2AX foci compared with the control (Fig. 3E, F). However, the percentages of cells with 53BP1 or γ-H2AX foci were significantly increased by EVs from irradiated hp-MSCs compared with the non-irradiated hp-MSCs (Fig. 3E, F). All these results indicated that irradiated hp-MSC EVs impaired the proliferation and induced DNA damage in HUVEC and H9c2 cells.

**Fig. 4:**
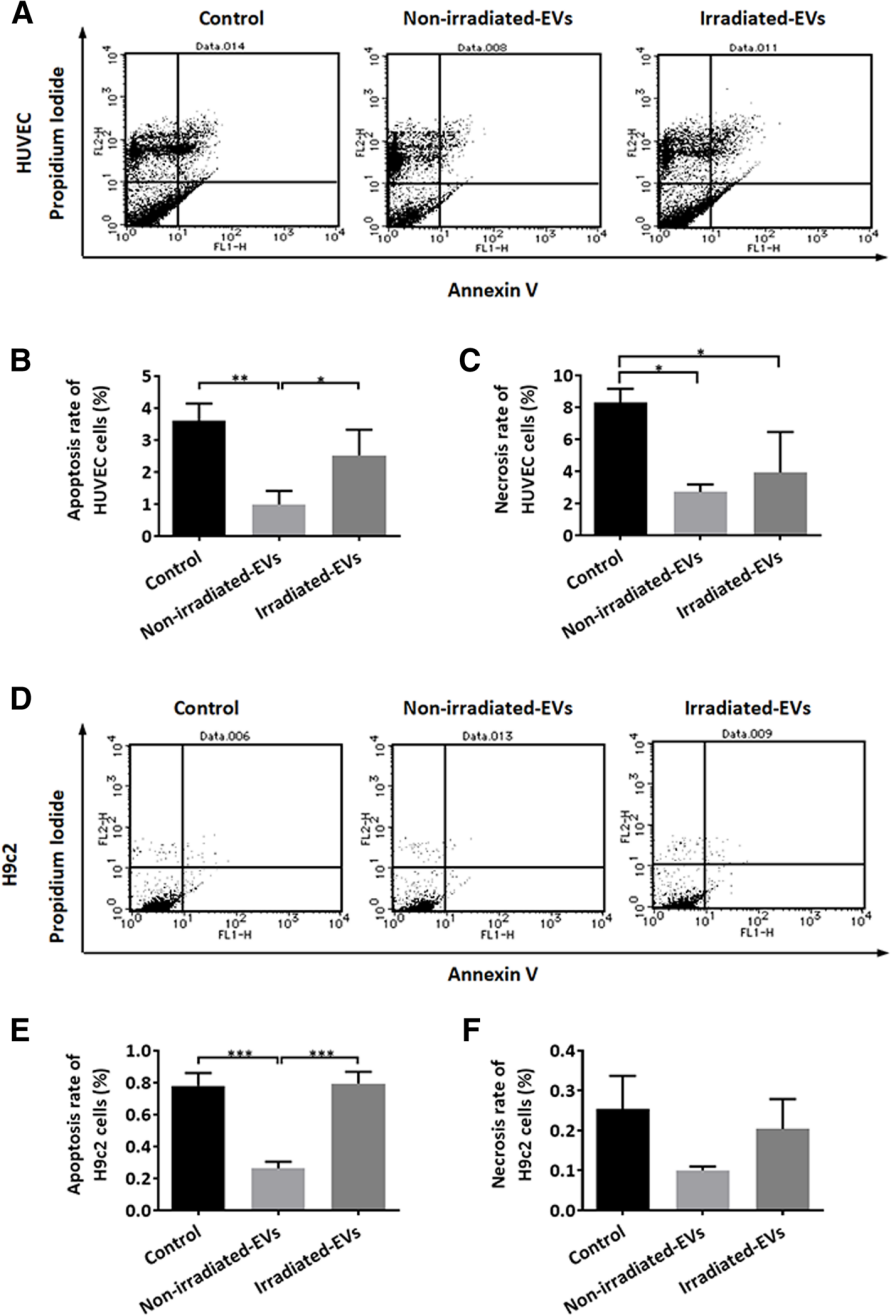
Effects of hp-MSC-derived EVs on the apoptosis of HUVEC and H9c2 cells. **A** Representative dot plots of flow cytometry analysis of the apoptosis of HUVEC cells. Quantitative data (*n*=3) on annexin-V-positive apoptotic HUVEC cells (**B**) and propidium iodide-labeled necrotic HUVEC cells (**C**). **D** Representative dot plots of flow cytometry analysis of the apoptosis of H9c2 cells. Quantitative data (*n*=3) on the apoptosis (**E**) and necrosis (**F**) of H9c2 cells. Non-irradiated-EVs: EVs isolated from conditioned medium of non-irradiated hp-MSCs, irradiated-EVs: EVs isolated from conditioned medium of irradiated hp-MSCs. Values are the mean ± SD. *P < 0.5, **P < 0.1, ***P < 0.01

Cell apoptosis was also evaluated after 48 h of coculture using an Annexin-V flow cytometry assay (Fig. 4A, D). The non-irradiated hp-MSC EVs significantly protected the HUVEC and H9c2 cells from apoptosis (Fig. 4 B, E). However, the apoptosis of HUVEC and H9c2 cells was less decreased by EVs from irradiated hp-MSCs than by EVs from non-irradiated hp-MSCs (Fig. 4B, E). Additionally, the necrosis of HUVEC cells was less decreased by EVs from irradiated hp-MSCs than by EVs from non-irradiated hp-MSCs (Fig. 4 C). However, neither the non-irradiated nor the irradiated hp-MSC EVs affected the necrosis of H9c2 cells (Fig. 4 F). In contrast to non-irradiated hp-MSC EVs, irradiated hp-MSC EVs showed a very poor ability to protect HUVEC and H9c2 cells from apoptosis.

### EVs from irradiated hp-MSCs revealed functional impairments to HUVEC and H9c2 cells

**Fig. 5:**
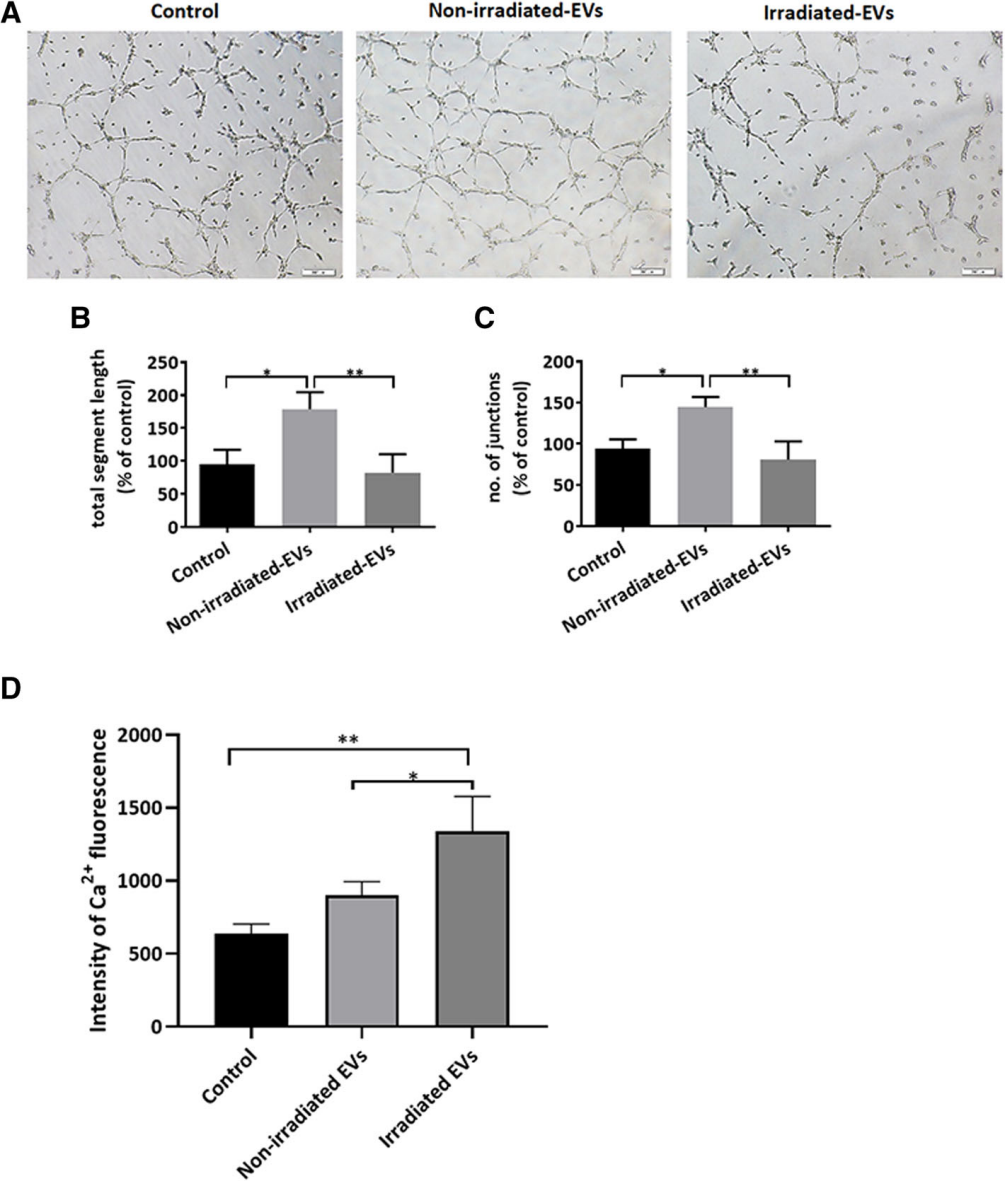
Effects of hp-MSC-derived EVs on tube formation of HUVEC cells and calcium transient of H9c2 cells. **A** Representative images of vessel-like structure formation on Matrigel®. HUVEC cells were previously cocultured with 10 μg/ml EVs from non-irradiated/irradiated hp-MSCs or not for 48 h. Scale bar: 200 μm. **B**, **C** Quantitative data (*n*=3) on tube formation relative to nontreated cells. **D** Quantitative analyses (*n*=3) of the calcium fluorescence intensity in H9c2 cells cocultured with 10 μg/ml EVs from non-irradiated/irradiated hp-MSCs or not for 48 h. Non-irradiated-EVs: EVs isolated from conditioned medium of non-irradiated hp-MSCs, irradiated-EVs: EVs isolated from conditioned medium of irradiated hp-MSCs. Values are the mean ± SD. ns *P* > 0.5, *P < 0.5, **P < 0.1

To evaluate the potential roles of EVs from irradiated hp-MSCs in cell function, we observed HUVEC cells tube formation (Fig. 5A) and calcium transient in H9c2 cells (Fig. 5D) 48 h after coculture. The tube formation of HUVEC cells was significantly increased by EVs from non-irradiated hp-MSCs but slightly decreased by EVs from irradiated hp-MSCs (Fig. 5B, C). However, the calcium transients of H9c2 cells were significantly increased by EVs from irradiated hp-MSCs, but were not changed significantly by EVs from non-irradiated hp-MSCs (Fig. 5D). These results indicated the functional impairment of irradiated hp-MSC EVs to HUVEC and H9c2 cells.

## Discussions

Emerging findings have identified the contribution of stem cell injury to radiation-induced tissue toxicity [23, 24]. EVs seem to be essential mediators of communication between MSCs and heart cells [25]. Resident MSCs in the heart are known to play an essential role in cardiac homeostasis [26], and their dysfunction may contribute to heart disease development [27]. Thus, following radiation exposure, the relatively highly radiosensitive MSCs may secrete specific EVs to induce injury to heart tissue cells, including cardiomyocytes and endothelial cells, which ultimately develop heart disease. To verify our hypothesis, we exposed hp-MSCs to 5 Gy γ-rays and then evaluated how EVs from irradiated hp-MSCs affect the biological properties of HUVEC and H9c2 cells.

We successfully isolated EVs from non-irradiated or irradiated hp-MSCs culture medium using ultracentrifugation, which was confirmed by electron microscopy and the expressions of CD63 and TSG101. As we isolated EVs for experiments before the publication of MISE2018 [22], we missed to examine the ratio of proteins:particles, lipids:particles, or lipids:proteins. Despite the similar amount and size distribution, EVs from non-irradiated and irradiated hp-MSCs revealed large differences in the expression of miRNAs, indicating radiation-induced alternation of EVs secreting MSCs in quality rather than in quantity.

MSC-derived EVs have been demonstrated to possess regenerative potential comparable to the regenerative potential of MSCs [28]. We next investigated the potential effects of EVs on heart tissue cells using HUVEC and H9c2 cells. Correspondingly, EVs from non-irradiated hp-MSCs clearly showed beneficial effects on cell proliferation, DNA damage, and cell apoptosis. In contrast, EVs from irradiated hp-MSCs revealed much less beneficial, and sometimes even harmful effects on HUVEC and H9c2 cells. We also aimed to identify whether EVs from irradiated hp-MSCs impair the functions of HUVEC and H9c2 cells. MSC-derived EVs promote angiogenesis by transferring signals to endothelial cells [29]. Tube formation of HUVEC cells was facilitated by EVs from non-irradiated hp-MSCs, but not irradiated hp-MSCs. The homeostasis of calcium transients is a crucial factor for maintaining normal cardiac rhythm [30]. Following ischemia/reperfusion injury, the internal levels of calcium in H9c2 cells increased in a time-dependent manner [31], and calcium overload in H9c2 cells may further accelerate reperfusion injury [32]. We found that calcium transients in H9c2 cells were significantly enhanced by EVs from only the irradiated hp-MSCs, indicating the induction of calcium overload in cells. All these data suggested the harmful, rather than beneficial effects of EVs from irradiated MSCs in heart cells.

Using miRNA sequencing, we found extensive changes in miRNAs between EVs from non-irradiated and irradiated hp-MSCs. Among the upregulated miRNAs in the EVs from irradiated hp-MSCs, the roles of miR-129-5p, miR-212-5p, miR-3120-5p, miR-16-1-3p, miR-4638-5p, miR-2392, and miR-324-3p have been reported mostly in cancer development, but rarely in cardiovascular diseases. Geng et al. found that high fat diet-induced upregulation of miR-129-5p contributes to atherosclerosis development via beclin-1 inhibition [33]. Zhao et al. identified that IgE activates miR-212-5p in asthmatic mice and causes decreased blood tension by downregulating vascular NCX1 expression [34]. Li et al. found that miR-3120-5p interacted with lncRNA WTAPP1 suppressing endothelial progenitor cell migration and angiogenesis by decreasing MMP-1 levels and inhibiting the PI3K/Akt/mTOR pathway [35]. Ge et al. reported that miR-324-3p promoted high glucose-induced renal fibrosis via activation of MAPK and ERK1/2 pathways [36]. Among the downregulated miRNAs in EVs from irradiated hp-MSCs, the roles of miR-4443, let-7a-5p, miR-382-5p, miR-424-3p, miR-3197, miR-3178, miR-32-3p, miR-1273g-3p, miR-23a-5p, miR-29a-3p, miR-146a-5p, miR-517a-3p, and miR-516b-5p have also been reported mostly in cancer progression. In addition, the roles of miR-1273g-3p, miR-23a-5p, miR-29a-3p, and miR-146a-5p in heart disease development have been studied broadly. Guo et al. determined that miR-1273g-3p promotes HUVEC cell dysfunction caused by acute glucose fluctuation [37]. miR-23a-5p enhances atherosclerotic plaque progression [38] and hepatic fibrosis [39, 40]. However, Lu et al. identified that miR-23a-5p was enriched in bone marrow-derived M2 macrophages with a reparative potential [41]. miR-29a-3p is known to reduce cardiac hypertrophy [42, 43] and ischemia-reperfusion injury [44]. miR-146a-5p is reported to attenuate ischemia/reperfusion injury by downregulating Irak1 and Traf6 and consequently blunting Toll-like receptor signaling [45]. miR-146a-5p deficiency in doxorubicin-treated mice leads to more severe cardiotoxicity [46]. Importantly, EVs from cardiosphere-derived cells have an abundant expression of miR-146a-5p, conferring cardiac regenerative therapeutic effects [47]. However, Oh et al. found that miR-146a-5p was enriched in extracellular vesicles isolated from failing hearts reducing cardiac contractility by suppressing SUMO1/SERCA2a signaling [48]. In contrast, Fang et al. found that patients with upregulated serum levels of miR-29a-3p and miR-146a-5p are more likely to develop diffuse myocardial fibrosis [49]. Thus, the exact roles of miR-23a-5p, miR-29a-3p, and miR-146a-5p need more in-depth investigation.

This study has several limitations (but is not limited to) that need further addressing. First, the culture scale of cardiac MSCs makes it difficult for us to obtain enough EVs for further experiments. As a proof-of-concept of study, we used MSCs from human placental tissues instead of the resident MSCs of the heart because of the availability of cell sources. MSCs are highly variable and heterogonous depending on source tissue and age. Therefore, it is of interest to estimate whether varied MSCs would respond differently to ionizing radiation. Second, hp-MSCs were exposed to only 5 Gy at a high dose rate (1 Gy/min) which is equivalent to the daily dose generally used in clinical radiotherapy for cancers. As the biological effects on cells vary greatly depending on the dose and dose rate of radiation [50, 51], it is necessary to evaluate the quantity and quality of EVs from MSCs by exposing the cells to different doses and dose rates of radiation. Third, we analyzed only miRNAs, but radiation exposure may also change other components such as lipids, proteins, and lncRNAs in EVs. Otherwise, we did not try to further confirm the role of each upregulated or downregulated miRNA in EVs from irradiated MSCs to HUVEC and H9c2 cells. Most importantly, we examined our hypothesis using only in vitro cell models, and animal models that mimic the in vivo changes after IR are worth studying. Finally, the pathological development of RIHD is complex, influenced by comprehensive factors released from all cells that resided in the heart (cardiomyocytes, endothelial cells, fibroblasts, stem cells). Herein, the interaction between these cells needed further investigation.

## Conclusions

Overall, although the number of EVs secreted from hp-MSCs was not changed by 5 Gy γ-ray exposure, EVs from irradiated hp-MSCs caused damage to HUVEC and H9c2 cells. Our preliminary data from an in vitro study demonstrated that EVs from MSCs may indirectly contribute to radiation-induced heart disease. Further studies, including interventional experiments and in vivo mouse models, are required to confirm our concept.

## Data Availability

All the datasets used and/or analyzed during this study are available from the corresponding authors on reasonable request.

## Abbreviations

MSCs: Mesenchymal stem cells
EVs: Extracellular vesicles
RT: Radiotherapy
hp-MSCs: Human placenta-derived mesenchymal stem cells
